# The Caenorhabditis elegans Ortholog of TDP-43 Regulates the Chromatin Localization of the Heterochromatin Protein 1 Homolog HPL-2

**DOI:** 10.1128/MCB.00668-17

**Published:** 2018-07-16

**Authors:** Tassa K. Saldi, Patrick Gonzales, Alfonso Garrido-Lecca, Vishantie Dostal, Christine M. Roberts, Leonard Petrucelli, Christopher D. Link

**Affiliations:** a Department of Molecular, Cellular, and Developmental Biology, University of Colorado, Boulder, Colorado, USA; b Integrative Physiology, University of Colorado, Boulder, Colorado, USA; c Department of Neuroscience, Mayo Clinic, Jacksonville, Florida, USA; d Institute for Behavioral Genetics, University of Colorado, Boulder, Colorado, USA

**Keywords:** ChIP-seq, RNA-seq, chromatin, dsRNA, neurodegeneration

## Abstract

TDP-1 is the Caenorhabditis elegans ortholog of mammalian TDP-43, which is strongly implicated in the etiology of frontotemporal dementia (FTD) and amyotrophic lateral sclerosis (ALS). We discovered that deletion of the *tdp-1* gene results in enhanced nuclear RNA interference (RNAi). As nuclear RNAi in C. elegans involves chromatin changes moderated by HPL-2, a homolog of heterochromatin protein 1 (HP1), we investigated the interaction of TDP-1 and HPL-2. We found that TDP-1 and HPL-2 interact directly and that loss of TDP-1 dramatically alters the chromatin association of HPL-2. We showed previously that deletion of the *tdp-1* gene results in transcriptional alterations and the accumulation of double-stranded RNA (dsRNA). These molecular changes are replicated in an *hpl-2* deletion strain, consistent with HPL-2 acting in consort with TDP-1 to modulate these aspects of RNA metabolism. Our observations identify novel mechanisms by which HP1 homologs can be recruited to chromatin and by which nuclear depletion of human TDP-43 may lead to changes in RNA metabolism that are relevant to disease.

## Abstract (precis)

TDP-1 is the Caenorhabditis elegans ortholog of mammalian TDP-43, which is strongly implicated in the etiology of frontotemporal dementia (FTD) and amyotrophic lateral sclerosis (ALS). We discovered that deletion of the *tdp-1* gene results in enhanced nuclear RNA interference (RNAi).

## INTRODUCTION

Pathological inclusions of the TDP-43 RNA binding protein are found in ∼97% of all amyotrophic lateral sclerosis (ALS) cases and ∼45% of all frontotemporal dementia (FTD) cases (1). The cytoplasmic TDP-43 inclusions observed in affected neurons are associated with reduced nuclear TDP-43 levels (2). Genetic and transgenic models in worms (3–5), flies (6, 7), zebrafish (8, 9), and mice (10–12) have implicated both gain-of-function and loss-of-function mechanisms in TDP-43 toxicity. Particularly strong support for the loss-of-function model comes from a recent study showing that partial loss of TDP-43 in all tissues in mice results in an ALS-like neurodegenerative phenotype (13). TDP-43 has been implicated in many components of RNA metabolism, including control of transcription, alternative splicing (AS), microRNA (miRNA) biogenesis, message stability, and formation of cytoplasmic RNA granules (1). Several recent reports also indicate that TDP-43 functions to limit the expression of endogenous retroviruses (14, 15), one of which (human endogenous retrovirus K) is overexpressed in ALS patients, likely contributing to neurodegeneration (16, 17). However, it is currently unclear which of these biological functions of TDP-43 are central to neurodegenerative pathology.

TDP-1 is the Caenorhabditis elegans ortholog of mammalian TDP-43: it has significant sequence similarity to TDP-43 in the RNA recognition motif (RRM) domains, binds the canonical TDP-43 binding sequence [(UG)_*n*_] with high affinity (18), and can substitute for human TDP-43 (hTDP-43) in *in vivo* splicing assays (3). We recently showed that deletion of TDP-1 results in the accumulation of double-stranded RNA (dsRNA) (19). This molecular phenotype is replicated when TDP-43 in the M17 human neuroblastoma cell line is knocked down by use of small interfering RNA (siRNA), suggesting that limiting the amount of dsRNA is a conserved function of TDP-1/TDP-43.

The accumulation of dsRNA in the TDP-1 mutant raised the possibility that this protein plays a role in the RNA interference (RNAi) pathway. In C. elegans, RNAi has been divided conceptually into two processes: exogenous RNAi (exo-RNAi), which is initiated by exposure to exogenous dsRNA, and endogenous RNAi (endo-RNAi), initiated by processing of endogenous transcripts (20). A competition between the exo- and endo-RNAi pathways exists in C. elegans and is believed to result from a functional overlap of RNAi factors between the two pathways (21). Indeed, both pathways require Dicer (DCR-1) for primary siRNA biogenesis as well as downstream effector proteins, such as Argonaute (Ago) proteins. Genetically removing components of the endo-RNAi pathway can result in a functional overexpression of some factors functioning in exo-RNAi, leading to increased efficiency of exo-RNAi (22). Exo- and endo-RNAi can act either in the cytoplasm, directing the RNAi-induced silencing complex (RISC) to target mRNAs for destruction, or in the nucleus, blocking transcription of targeted sequences. The latter process is termed nuclear RNAi or transcriptional gene silencing (TGS). TGS is mediated by siRNAs brought to the site of transcription by the nuclear RNAi-deficient (NRDE) complex. This results in the inhibition of transcription at targeted loci followed by heterochromatin formation, likely facilitated by the subsequent recruitment of the C. elegans heterochromatin protein 1 (HP1) homolog HPL-2 (reviewed in reference 23).

Along with HP1/HPL-2's role in transcriptional gene silencing, HP1 homologs have also been shown to function in a variety of other processes, including chromatin organization, DNA replication, and the DNA damage response (24). Additionally, recent studies of humans and flies showed that HP1 is localized to areas of active transcription and associates with both gene bodies and promoters (25, 26). The Drosophila HP1 homolog associates with genes in an RNA-dependent manner and copurifies with several pre-mRNA processing factors, including hnRNP proteins (27, 28), suggesting a role for HP1 in pre-mRNA processing. Consistent with this idea, HP1 in humans modulates both mRNA abundance and pre-mRNA splicing (26, 29).

Recent work with C. elegans indicates that the HP1 homolog, HPL-2, also binds to highly transcribed genes and modulates mRNA abundance (30), as well as binding and repressing repetitive elements (31). Interestingly, while HP1 homologs directly bind H3K9me2/3 histone modifications via a chromodomain (32, 33), H3K9me2/3 is not endogenously required in C. elegans for HPL-2 association (30). While several C. elegans genes have been suggested to be involved in HPL-2 recruitment, including *lin-13*, *lin-35*, and *let-418* (34–36), the mechanism of HPL-2 association with active genes is unclear. In humans, HP1 coprecipitates with elongating forms of RNA polymerase II (Pol II) (26) but only localizes to certain genes to affect RNA processing, implying that additional factors must provide the specificity of HP1 association.

Here we show that loss of TDP-1 sensitizes C. elegans to somatic exogenous RNAi and that this effect is dependent on the nuclear RNAi process. Synthetic phenotypes of animals mutant for both *tdp-1* and an essential component of the nuclear RNAi complex, *nrde-3*, suggest that TDP-1 functions in parallel to TGS. Because nuclear RNAi invokes chromatin changes, we asked if TDP-1 interacts with the C. elegans HP1 homolog, HPL-2. We found that TDP-1 coimmunoprecipitates with HPL-2 in a manner that is independent of endogenous siRNAs (endo-siRNAs) and that TDP-1 facilitates HPL-2 association with active genes to maintain mRNA abundance. This novel function of TDP-1 may explain the HPL-2 specificity for a subset of genes and may have implications for the molecular functions of TDP-43 that are relevant to human disease.

## RESULTS

### Loss of TDP-1 enhances exogenous RNAi by enhancing nuclear RNAi.

**FIG 1:**
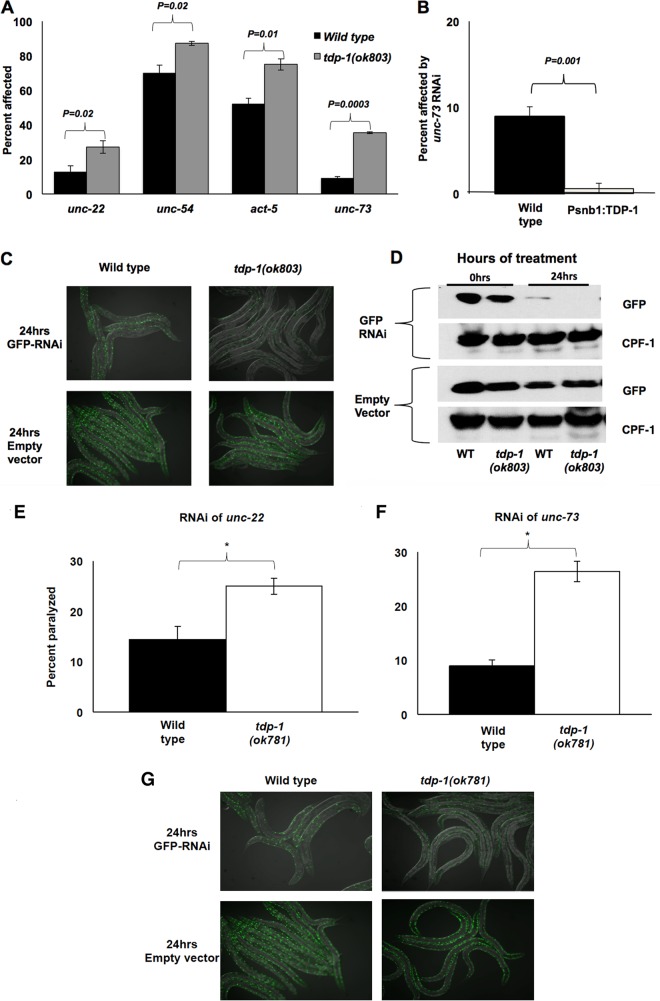
TDP-1 limits the efficiency of exogenous RNAi. (A) Percentages of animals affected by treatment with RNAi against the indicated genes. (B) Suppression of neuron-specific *unc-73* RNAi by transgenic pan-neuronal overexpression of TDP-1. (C) Representative pictures of GFP fluorescence in wild-type and *tdp-1*(*ok803*) animals expressing GFP driven by a *myo-3* (muscle-specific) promoter that were treated with GFP feeding RNAi for 24 h. Treatment was done two independent times. (D) Western blots of total proteins from wild-type (WT) and *tdp-1*(*ok803*) GFP-expressing animals before and after 24 h of GFP RNAi (top two panels) or treatment with empty vector (bottom two panels). CPF-1 is a loading control. (E and F) Percentages of wild-type and *tdp1*(*ok781*) animals affected by RNAi of the indicated genes. (G) Representative images of wild-type and *tdp-1*(*ok781*) animals expressing GFP driven by a *myo-3* (muscle-specific) promoter that were treated with GFP RNAi or empty vector for 24 h. For panels A, B, E, and F, RNAi assays were performed three independent times (in triplicate), with >100 animals scored per plate. *P* values across three biological replicates were calculated by Student's *t* test; error bars show standard errors of the means (SEM).

To assay the effect of TDP-1 on RNAi, we grew *tdp-1* null worms [*tdp-1*(*ok803*)] and wild-type worms on bacteria producing dsRNAs against a variety of somatic genes. Quantification of the percentage of progeny affected by each type of RNAi indicated that *tdp-1* mutants had a mild but reproducible increased sensitivity to exo-RNAi (Fig. 1A). Conversely, transgenic worms with neuronal overexpression of TDP-1 (P*snb-1*::TDP-1) were highly resistant to neuron-specific *unc-73* RNAi: less than 1% of treated animals displayed an Unc phenotype, compared to ∼10% of wild-type animals (Fig. 1B). This result is in line with a recent report showing that overexpression of human TDP-43 in the Drosophila central nervous system (CNS) also decreased RNAi efficiency (15), suggesting that the effect of *tdp-1* on RNAi is a conserved function of the encoded protein.

**FIG 2:**
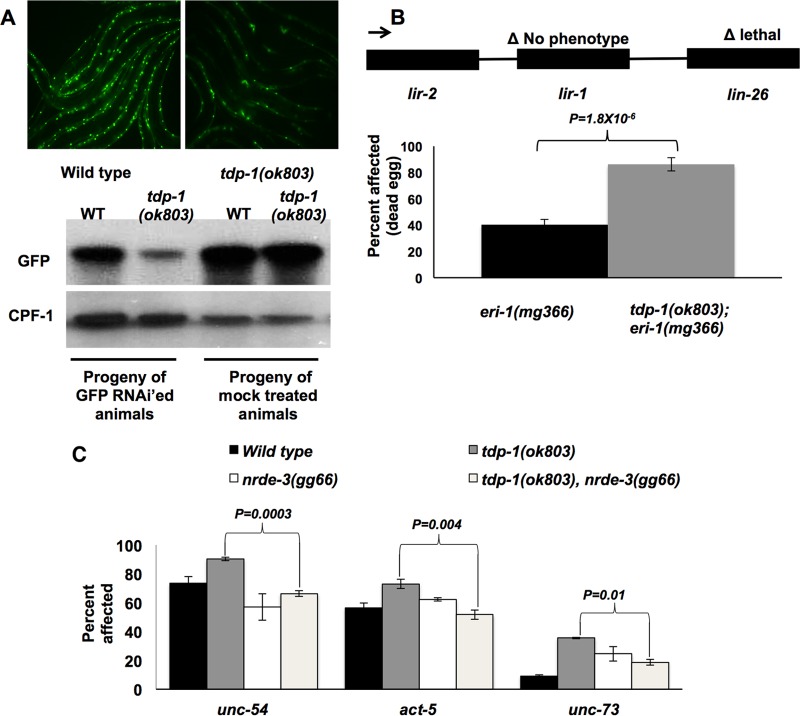
TDP-1 limits RNAi by limiting nuclear RNAi. (A) (Top) Representative examples of GFP fluorescence in the untreated progeny (L4) of GFP RNAi-treated parents expressing a *myo-3*::GFP transgene in wild-type and *tdp-1*(*ok803*) backgrounds. (Bottom) Western blots of total proteins from 48-h-old untreated progeny of wild-type and *tdp-1*(*ok803*) animals treated with GFP feeding RNAi or empty vector. CPF-1 is a loading control. (B) Percentages of animals affected by *lir-1* feeding RNAi. A schematic of the operon containing *lir-1* and *lin-26* is shown at the top. Boxes indicate genes, and the line represents the intercistronic space. The *P* value was calculated by Student's *t* test. (C) Loss of NRDE-3 function reverses the enhanced RNAi efficiency of the *tdp-1*(*ok803*) mutant. Genes targeted by RNAi in the indicated strains are shown on the *x* axis. *P* values were calculated by comparing values for the *tdp-1*(*ok803*) mutant alone to those for the *tdp-1*(*ok803*); *nrde-3*(*gg66*) double mutant by Student's *t* test. In panels B and C, error bars show SEM across three biologically independent replicates (done in triplicate), with >100 animals assayed per plate.

To rule out the possibility that *tdp-1* mutant animals were sensitive to RNAi due to gene-specific effects, we assayed for RNAi sensitivity to a nonendogenous gene. Wild-type and *tdp-1*(*ok803*) mutant animals expressing muscle-specific green fluorescent protein (GFP) from an integrated transgene were subjected to GFP-specific RNAi by feeding (GFP feeding RNAi) or treated with an empty vector. Comparison of the GFP signals following 24 h of treatment indicated that RNAi against GFP was more effective in *tdp-1* mutants than in wild-type animals, mirroring the results seen for endogenous genes (Fig. 1C and D). Analysis of the nonnull allele of *tdp-1*, *tdp-1*(*ok781*), showed similar results (Fig. 1E to G). Interestingly, we noticed that the L4 and adult progeny of the *tdp-1* mutant treated with GFP RNAi showed a much more dramatic knockdown than that of the progeny of the wild-type control. These results led us to suspect that *tdp-1* mutant animals had heightened sensitivity to nuclear RNAi, as nuclear RNAi is required to maintain heritable gene silencing in the progeny of treated animals (37). We confirmed this by transferring embryos of *tdp-1* mutant and wild-type worms treated for 1 generation with GFP RNAi to normal (Escherichia coli OP50) bacteria and then assaying GFP expression of these progeny at the L4 stage, as done previously (37). As expected, wild-type progeny of treated animals showed a mild decrease in GFP fluorescence compared to that for untreated controls (Fig. 2A). Strikingly, *tdp-1*(*ok803*) progeny displayed a dramatic reduction in GFP levels, with some animals displaying no observable GFP fluorescence (Fig. 2A), and this was confirmed by immunoblotting (Fig. 2A, bottom panel).

Pronounced sensitivity to heritable gene silencing in *tdp-1* mutants may be explained if TDP-1 limits the efficiency of nuclear RNAi. We tested if *tdp-1* mutants were specifically sensitive to nuclear RNAi by targeting genes transcribed in operons (polycistronic gene clusters driven by a common promoter). Because nuclear but not cytoplasmic RNAi results in inhibition of elongating RNA Pol II (38), nuclear RNAi targeting of an upstream gene in an operon causes knockdown of all the downstream transcripts within that operon. This effect can be assayed in C. elegans by measuring lethality induced by RNAi of *lir-1*, a nonessential gene upstream of *lin-26*, an essential gene in the same three-gene operon (39). We found that loss of TDP-1 increased the lethality of *lir-1* RNAi >2-fold (Fig. 2B), demonstrating that *tdp-1* mutants have a dramatic sensitivity to nuclear RNAi. Further, we found that the sensitivity of *tdp-1*(*ok803*) animals to exo-RNAi was due to enhanced nuclear RNAi, as introduction of the *nrde-3*(*gg66*) mutation into the *tdp-1*(*ok803*) strain completely reversed the enhanced RNAi sensitivity (Fig. 2C). We did note that the *nrde-3* mutant showed mild sensitivity to neuronal RNAi (*unc-73*). However, mutation of *nrde-3* in the *tdp-1*(*ok803*) background reversed the sensitivity to *unc-73* RNAi, to the level of the *nrde-3* mutant alone, supporting the conclusion that enhanced sensitivity to *unc-73* RNAi in *tdp-1* mutants is dependent on nuclear RNAi.

### TDP-1 is not required for expression of endogenous siRNAs.

Mutation of C. elegans genes involved in production of endo-siRNAs can result in enhanced exo-RNAi sensitivity. Therefore, we considered that *tdp-1* mutants might be sensitive to nuclear exo-RNAi due to *tdp-1* playing a role in the production of endo-siRNAs. To address this possibility, we deep sequenced small RNAs from *tdp-1*(*ok803*) and wild-type animals to look for decreases in endo-siRNA abundance in the mutants. We created ligation-independent small RNA sequencing libraries to capture molecules with either a 5′ monophosphate (primary siRNAs, microRNAs, and PIWI-interacting RNAs [piRNAs]) or a 5′ triphosphate (secondary siRNAs).

**FIG 3:**
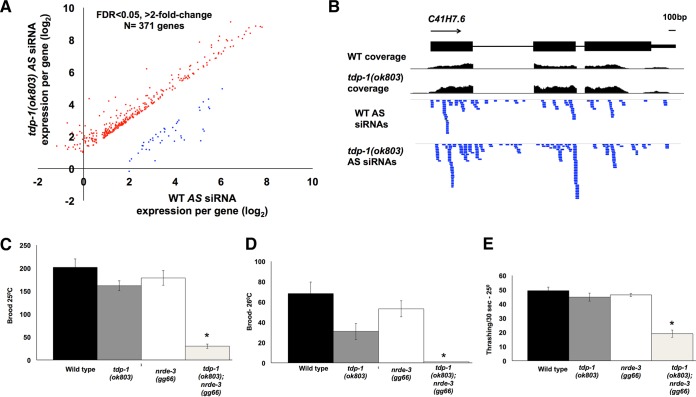
*tdp-1* and *nrde-3* function redundantly to maintain health. (A) Fold changes (log_2_) in abundance of antisense (AS) siRNAs mapping to annotated genes between wild-type and *tdp-1*(*ok803*) mutants. Each dot represents an individual gene with an increased (red dots) or decreased (blue dots) abundance of AS siRNAs targeting that gene. (B) Example of a gene with increased antisense siRNA abundance in *tdp-1*(*ok803*) animals compared to that in wild-type animals. Blue boxes represent AS siRNA reads mapping to *C41H7.6* in each sample. Coverage tracks are normalized to the total number of reads. (C and D) Average brood sizes for the indicated strains grown at 25°C (C) and 26°C (D). Broods were counted for at least 5 animals per strain, in triplicate, two independent times. Note that *tdp-1*(*ok803*); *nrde-3*(*gg66*) mutants are completely sterile at 26°C. (E) Number of thrashes counted per 30 s for each of the indicated strains. Also see Movies S1 and S2 in the supplemental material. Animals were grown from embryos at 25°C and scored as first-day adults. Thrashing was counted in at least 10 animals, in triplicate, two independent times. In panels C to E, error bars show SEM. *, *P* < 0.01 (calculated using one-way analysis of variance [ANOVA] with Tukey's highly significant difference [HSD] *post hoc* test).

The small RNA libraries yielded an average of ∼10 million uniquely mapped reads per library (mutant and wild-type libraries were created in duplicate). To identify siRNA target genes, the sequences were filtered for reads mapping antisense to annotated genes (excluding piRNAs and microRNAs). We identified ∼6,000 genes targeted by antisense siRNAs, in good agreement with the results of previous studies (40). Direct comparisons of the abundances of antisense siRNAs across targeted genes by use of DESeq software indicated that 372 genes had significantly different (false-discovery rate [FDR] of <0.05; >2-fold change) siRNA abundances between the wild type and the *tdp-1* mutant (see Data Set S1 in the supplemental material). Consistent with our previous finding that TDP-1 limits the abundance of dsRNA, which is the precursor to siRNAs, the large majority (>88%) of genes with a differential abundance of antisense siRNAs contained increased levels in *tdp-1*(*ok803*) animals compared to those in wild-type animals (Fig. 3A). Figure 3B shows an example of a gene with increased targeting by siRNAs in *tdp-1* mutants. This result indicates that TDP-1 is not required to maintain abundance for the majority of siRNAs but instead suggests that TDP-1 functions in limiting the steady-state level of some siRNAs. Furthermore, we saw no enrichment for increased or decreased levels of siRNAs mapping to NRDE-3 target genes (χ^2^ = 0.14) (target genes were obtained from reference 41), indicating that TDP-1 does not play a specific role in the production of NRDE-3-bound siRNAs.

### TDP-1 and NRDE-3 redundantly maintain health at elevated temperatures.

As TDP-1 was not required for the production of endo-siRNAs, we asked if *tdp-1* associated with the nuclear RNAi/NRDE complex to limit nuclear RNAi. However, we were unable to detect an RNA-independent association between TDP-1 and NRDE-3 in the lysate of a strain expressing N-terminally FLAG-tagged NRDE-3 by coimmunoprecipitation with anti-TDP-1 or anti-FLAG antibodies (41; data not shown), suggesting that TDP-1 and NRDE-3 do not associate in a common complex. Furthermore, comparison of genes cotranscriptionally bound by TDP-1 (taken from reference 19) and genes targeted by NRDE-3-associated siRNAs (taken from reference 41) indicated that only 7 of the 173 known NRDE-3 target genes were also bound by TDP-1, providing additional evidence that TDP-1 does not function with the NRDE complex on the majority of NRDE target genes.

If *tdp-1* does not maintain endo-siRNA production or associate with the NRDE complex, why are *tdp-1* mutant animals sensitive specifically to nuclear RNAi? One possibility is that *tdp-1* functions in a parallel pathway that utilizes one or more factors also involved in nuclear RNAi. In this situation, the absence of *tdp-1* would increase the availability of the shared factor(s), resulting in increased nuclear RNAi efficiency, analogous to the competition between endo- and exo-RNAi (see the introduction). To ask if *tdp-1* functions in parallel to the NRDE complex, we asked if *tdp-1*(*ok803*); *nrde-3*(*gg66*) double mutants showed synthetic phenotypes. While we observed no obvious defects in *tdp-1*(*ok803*); *nrde-3*(*gg66*) animals at a normal growth temperature (20°C), shifting the animals to 25°C resulted in a maternal-effect decrease in brood size (Fig. 3C) and delayed fertility (∼24-h delay) (data not shown). At 26°C, *tdp-1*(*ok803*); *nrde-3*(*gg66*) animals showed complete maternal-effect sterility (Fig. 3D). Additionally, *tdp-1*(*ok803*); *nrde-3*(*gg66*) double mutants grown from embryos at 25°C had clearly uncoordinated movement (Unc phenotype) and showed an approximately 2-fold decrease in thrashing rate compared to that for the wild type and/or each of the single mutants (Fig. 3E; Movies S1 and S2). These results indicated that *tdp-1* does not function through or with *nrde-3* but likely functions redundantly, in a parallel pathway, to maintain fertility and normal movement. The synergistic effects observed for the *tdp-1*; *nrde-3* double mutant appeared to be specific, as we did not note a genetic interaction between *tdp-1*(*ok803*) and loss-of-function mutations in other chromatin factors, including *set-2*, *met-2*, *lin-35*, and *hpl-2* (data not shown).

### TDP-1 associates directly with HPL-2.

**FIG 4:**
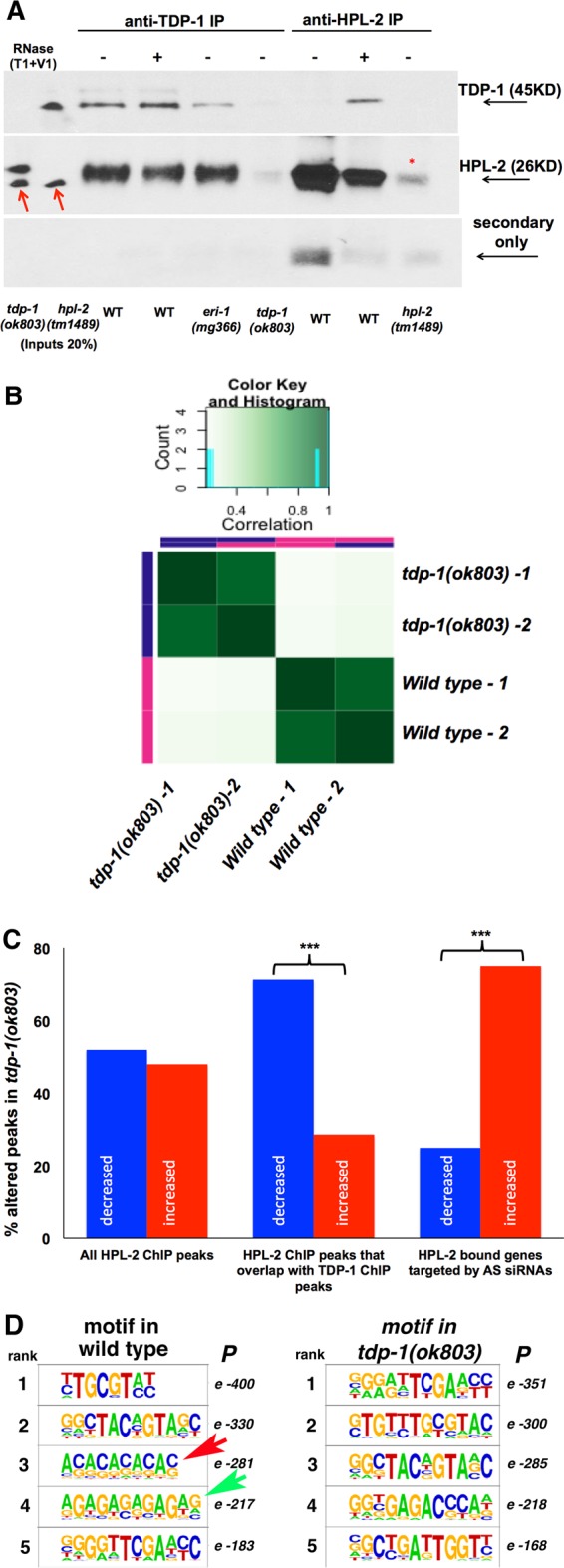
HPL-2 interacts directly with TDP-1. (A) Western blot probed with anti-TDP-1 (top panel) and anti-HPL-2 (middle panel) antibodies against protein immunoprecipitated with either anti-TDP-1 antibodies (lanes 3 to 6) or anti-HPL-2 antibodies (last three lanes) in extracts prepared from the indicated strains. IPs were performed with null *tdp-1*(*ok803*) and *hpl-2*(*1489*) mutant strains to control for nonspecific protein associations. The size of each protein is indicated. Note that HPL-2 runs slightly above the light chain band (∼26 kDa), even though HPL-2 is predicated to run at 21 kDa. The bottom panel was probed with secondary antibody only to show light chain-specific contamination. Treatment with both T1 (single-stranded RNA specific) and V1 (double-stranded RNA specific) RNases is indicated. Red arrows indicate a nonspecific background band recognized by the HPL-2 antibody used as an internal loading control. *, light chain contamination. (B) Correlation heat map showing high similarity between wild-type and *tdp1*(*ok803*) HPL-2 ChIP-seq replicates. Darker green shading indicates increased similarity. (C) Percentages of peaks in the indicated categories that were either increased or decreased for HPL-2 localization in *tdp-1*(*ok803*) mutants. ***, *P* < 1 × 10^−10^ (chi-square test). (D) Enriched binding motifs identified in HPL-2 ChIP-seq analyses of wild-type (left) and *tdp-1*(*ok803*) (right) animals, arranged according to decreasing *P* value. Sequences were filtered to keep the top five (by *P* value) unique motifs longer than 7 nt for both wild-type and *tdp-1*(*ok803*) animals. Red and green arrows indicate binding motifs identified in wild-type but not *tdp-1*(*ok803*) HPL-2 ChIP-seq experiments.

Nuclear RNAi results in reduced RNA Pol II occupancy downstream of regions targeted by siRNAs and in deposition of repressive heterochromatin marks (38, 42). HPL-2 has been shown to be recruited to regions undergoing nuclear RNAi and promotes transcriptional repression at these loci (43). However, HPL-2 likely also functions independently of nuclear RNAi, as *hpl-2* deletion results in maternal-effect, temperature-sensitive sterility not observed in *nrde-3*(*gg66*) mutants (34). Considering that *tdp-1*(*ok803*); *nrde-3*(*gg66*) double mutants also displayed temperature-sensitive sterility, we hypothesized that *tdp-1* may function in parallel to the NRDE complex to control HPL-2 function/recruitment. To determine if TDP-1 could modulate HPL-2 availability or chromatin association by a direct interaction, we asked if TDP-1 antibodies could immunoprecipitate HPL-2. As shown in Fig. 4A, TDP-1 was able to immunoprecipitate HPL-2 in both the presence and absence of RNA. TDP-1 also immunoprecipitated HPL-2 in an *eri-1* mutant background. As loss of *eri-1* blocks endo-siRNA production and subsequent NRDE-3 nuclear localization (41), this result indicates that TDP-1 immunoprecipitated HPL-2 even in the absence of somatic endo-siRNAs and nuclear NRDE-3. In the reciprocal experiment, HPL-2 also immunoprecipitated the TDP-1 protein (Fig. 4A, last 3 lanes). However, this association was observed only following the addition of RNase to the extract, possibly suggesting that the epitope recognized by the HPL-2 antibody is hidden when HPL-2 is in complex with both TDP-1 and RNA. Alternatively, this result may reflect a differential abundance of free TDP-1 and free HPL-2 relative to that of the TDP-1/HPL-2 complex.

### TDP-1 maintains HPL-2 association in gene bodies.

To ask if the association of TDP-1 with HPL-2 is needed for HPL-2 recruitment to chromatin, we performed a chromatin immunoprecipitation (ChIP) assay with an antibody against HPL-2, followed by deep sequencing, for *tdp-1*(*ok803*) mutants and wild-type animals. Using the model-based analysis of ChIP-seq (MACS) peak-calling algorithm, we identified ∼14,000 distinct peaks of HPL-2 association in either or both wild-type and *tdp-1*(*ok803*) animals (Data Set S2), which showed high reproducibility (Fig. 4B). As previously observed by other researchers (30, 31), the large majority of HPL-2 peaks for wild-type animals overlapped annotated repetitive elements (73%), and our called HPL-2 ChIP peaks also significantly (*P* < 0.001; *Z* > 96) overlapped peaks previously identified by McMurchy et al. (31), as determined by permutation tests (see Materials and Methods). Having validated our HPL-2 ChIP peaks, we then used the Diffbind algorithm to identify 8,813 regions with statistically significantly different (*P* < 0.05; FDR < 0.1) HPL-2 associations between *tdp-1*(*ok803*) mutants and wild-type animals, with roughly equal numbers of regions with decreased and increased HPL-2 associations in the mutant (Data Set S2).

In order to focus on HPL-2-bound regions likely to be affected directly by TDP-1, we filtered differentially bound HPL-2 ChIP peaks for regions also directly associated with TDP-1 (15). Remarkably, 50% of previously identified TDP-1 ChIP peaks (2,777/5,579 peaks) overlapped HPL-2 peaks differentially bound in *tdp-1*(*ok803*) mutants (Data Set S3), the majority of which (71%) showed a decrease in HPL-2 association in the mutant compared to that in the wild type (Fig. 4C). This highly significant enrichment for decreased association of HPL-2 in these regions (*P* = 1.25 × 10^−63^; chi-square test) indicates that in the majority of regions where TDP-1 affects HPL-2 localization, TDP-1's role is to maintain HPL-2's chromatin association.

To ask if TDP-1-mediated localization of HPL-2 was facilitated by endo-siRNAs (analogous to NRDE-3 recruitment of HPL-2), we asked if genes with *tdp-1*-dependent HPL-2 association were also siRNA target genes (taken from this study). However, genes with TDP-1-dependent HPL-2 peaks showed no enrichment for targeting by endo-siRNAs (*P* = 0.3; chi-square test), nor did we find an enrichment for genes with altered siRNA abundance in *tdp-1* mutants among genes with *tdp-1*-dependent HPL-2 ChIP peaks (*P* = 0.1; chi-square test). These results suggest that TDP-1-mediated HPL-2 localization is likely not facilitated by siRNAs. Interestingly, examination of HPL-2 ChIP signals for all siRNA target genes, regardless of colocalization with TDP-1, revealed a strong enrichment for siRNA target genes with increased HPL-2 ChIP signals in *tdp-1* mutants (*P* = 1.6 × 10^−29^; chi-square test) (Fig. 4C, data set on the right), indicating that *tdp-1* indirectly limits HPL-2 localization to siRNA target genes. This result is unlikely to be due solely to increased levels of endo-siRNAs in *tdp-1* mutants, as only 2% of siRNA target genes with increased HPL-2 ChIP signals also showed higher siRNA abundances in *tdp-1*(*ok803*) animals. Taken together, these observations support the ideas that TDP-1 maintains HPL-2 localization in an siRNA-independent manner and that deletion of *tdp-1* leads to increased availability of HPL-2 to siRNA target genes and the nuclear RNAi complex.

### HPL-2 binds the TDP-1 binding motif in a *tdp-1*-dependent manner.

TDP-1 localization to chromatin is dependent on RNA (15), and TDP-1 is required for HPL-2 association at many locations. These observations support the idea that TDP-1 may directly bind nascent RNA and recruit HPL-2 to or maintain HPL-2 localization at TDP-1-bound genes. If this model is correct, it predicts that HPL-2 should localize to the previously characterized TDP-1 consensus binding motif, (UG)_*n*_ [(TG)_*n*_/(AC)_*n*_ in the DNA] (18). To investigate this idea, we used HOMER software (see Materials and Methods) to identify enriched binding motifs among HPL-2-bound regions in wild-type animals (Fig. 4D, left panel). Indeed, an (AC)_*n*_ repeat [(TG)_*n*_ on the opposite strand] (Fig. 4D, red arrow) was the third most significant binding motif identified. This motif was present in over 25% of all regions bound by HPL-2. Importantly, an identical analysis of enriched HPL-2 binding motifs in *tdp-1*(*ok803*) mutants failed to identify (AC)_*n*_ repeats as bound by HPL-2 (Fig. 4D, right panel), indicating that TDP-1 is likely responsible for mediating HPL-2 localization to (AC)_*n*_/(TG)_*n*_ repeats. Interestingly, we noted that a second binding motif, (AG)_*n*_, identified for HPL-2 in wild-type animals (Fig. 4D, green arrow), was also not enriched in *tdp-1* mutants. While (AG)_*n*_ repeats have not previously been characterized as a binding site for TDP-43 orthologs, it is possible that TDP-1 associates with another RNA binding protein specific to this site in order to recruit/maintain HPL-2 at chromatin (see Discussion). Regardless, (AC)_*n*_ and (AG)_*n*_ motifs were identified in over 50% of all regions bound by HPL-2 in wild-type animals, indicating that TDP-1 mediates specificity for the majority of HPL-2 binding locations.

### TDP-1-dependent HPL-2 recruitment maintains transcript abundance.

**FIG 5:**
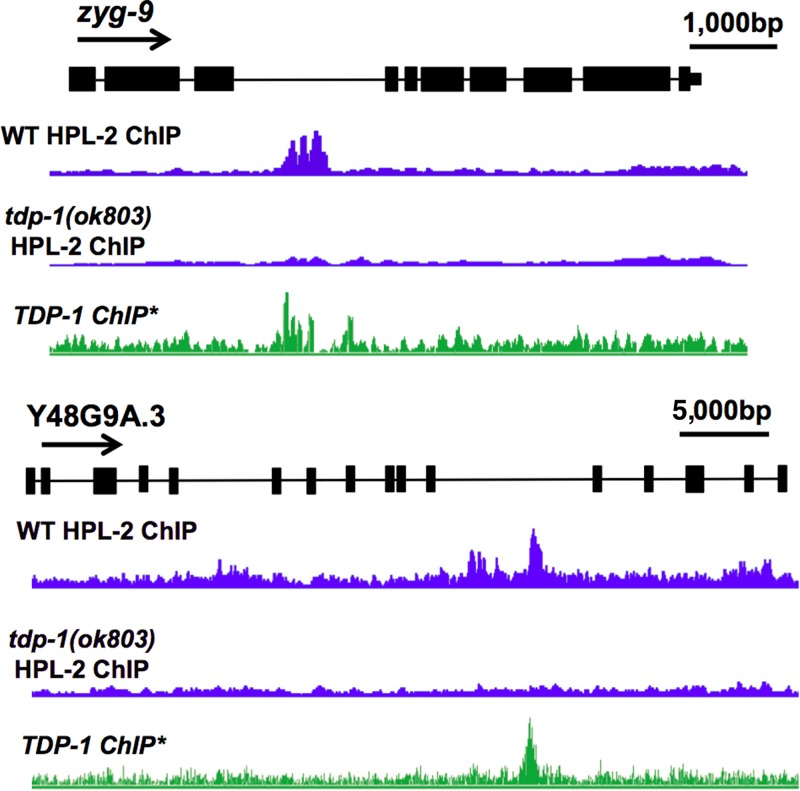
TDP-1-dependent recruitment of HPL-2 to chromatin. Examples of introns that have TDP-1-dependent HPL-2 ChIP-seq peaks are shown. For each track, the coverage is normalized to the total number of reads. *, data were taken from reference 15 (accession number GSE61581).

To determine what types of HPL-2-bound regions are mediated by TDP-1, we compared the locations of differentially bound HPL-2 peaks associated with TDP-1 to the locations of annotated genes. Interestingly, we found a significant enrichment of HPL-2/TDP-1 peaks in gene bodies compared to differentially bound HPL-2 peaks not associated with TDP-1 (82% bound by TDP-1 compared to 65% not bound [*P* = 2.68 × 10^−48^; chi-square test]). Specifically, we noted that the locations of these TDP-1-dependent HPL-2 ChIP peaks corresponded to intronic regions (71%) and promoters (20%). Indeed, TDP-1/TDP-43 orthologs have been shown to bind predominantly to introns in multiple organisms (44, 45). Two examples of TDP-1-bound introns with reduced HPL-2 association in *tdp-1*(*ok803*) animals compared to that in wild-type animals are shown in Fig. 5.

HP1 controls the abundance of transcripts from active genes (26, 28–30). To ask if TDP-1-dependent HPL-2 recruitment also affects gene expression, we compared mRNA abundances between *tdp-1*(*ok803*) and wild-type animals for genes with TDP-1-dependent HPL-2 ChIP peaks. Of the 1,005 genes with TDP-1-dependent HPL-2 peaks that were expressed in our mRNA data sets, 161 showed a significantly changed RNA abundance in *tdp-1* mutant animals compared to that in wild-type animals. Importantly, 137 of these genes (85%) showed decreased transcript abundance, representing a highly significant enrichment for decreased expression. This includes all transcripts altered for expression in *tdp-1* mutants (*P* = 5.7 × 10^−22^; chi-square test) and transcripts with altered expression from genes identified as cotranscriptionally bound by TDP-1 (*P* = 2.9 × 10^−5^; chi-square test). This result is consistent with the hypothesis that when TDP-1 binds nascent RNA in concert with HPL-2, a function of this complex is to maintain transcript abundance.

### Deletion of *hpl-2* replicates some molecular phenotypes of *tdp-1* deletion.

**FIG 6:**
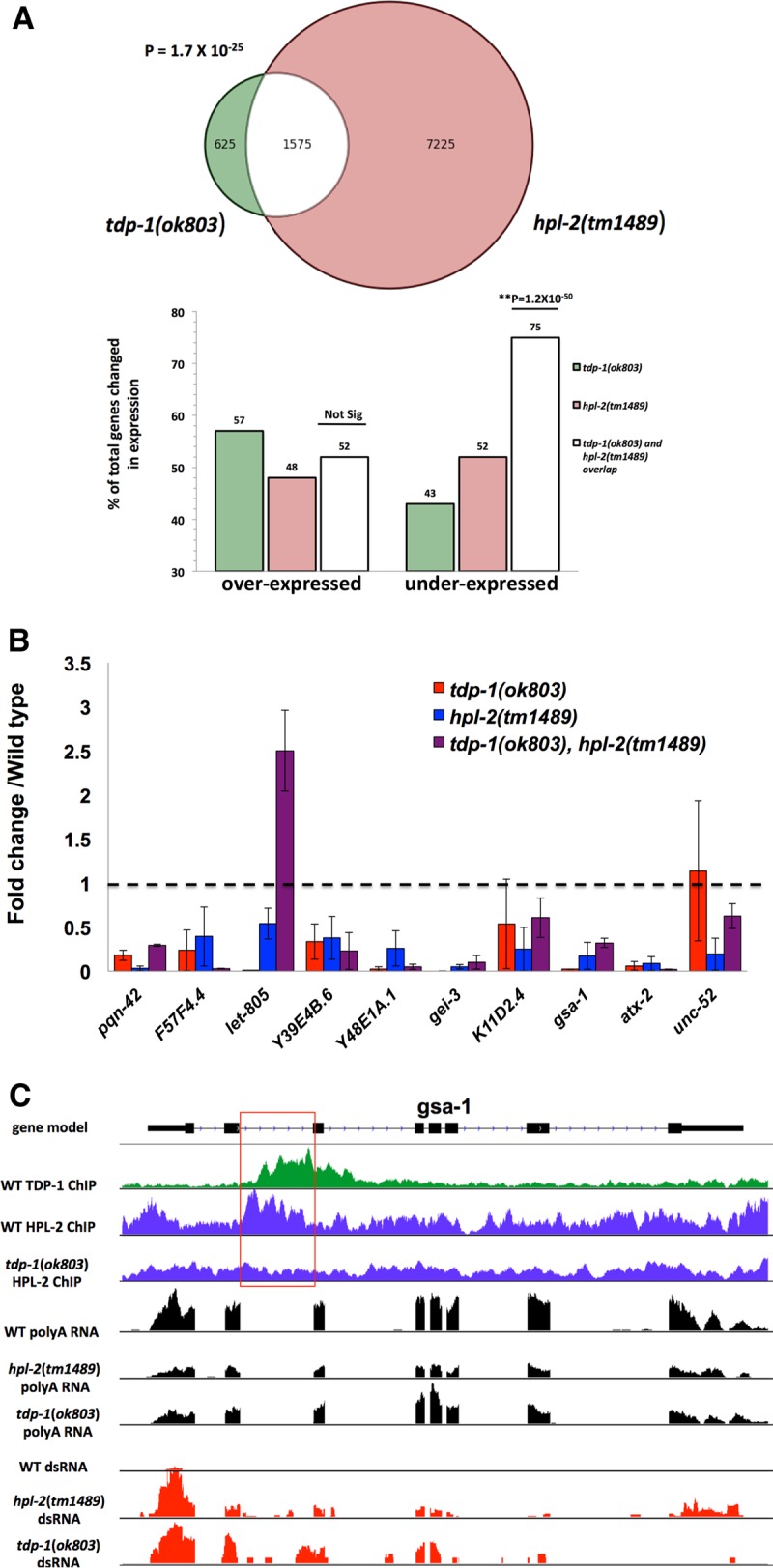
Overlapping transcriptome changes in *tdp-1* and *hpl-2* deletion mutants. (A) Venn diagram representing overlapping transcriptional changes in *tdp-1*(*ok803*) and *hpl-2*(*tm1489*) mutants (top) and bar graph illustrating that transcripts altered by both mutations are preferentially underexpressed (bottom). (B) Quantitative RT-PCR confirmation of selected transcripts with reduced accumulation in the *tdp-1* and *hpl-2* mutants relative to that in the wild type as identified by RNA-seq. For each transcript, primers were designed to target an exon, and amplification levels were normalized to the transcript level of *pmp-3*, a gene whose expression is not affected by the mutations tested. (C) Effects of TDP-1 and HPL-2 on the representative gene *gsa-1*. The green histogram displays ChIP-seq data for TDP-1 (15) (accession number GSE61581). Note the peak of TDP-1 binding localized over the second intron. Blue histograms display HPL-2 ChIP-seq data for wild-type (upper track) and *tdp-1*(*ok803*) (lower track) worms. Note the peak of HPL-2 binding in the second intron, which was lost in the *tdp-1* deletion mutant. Black histograms display *gsa-1* poly(A) transcript accumulation in wild-type, *hpl-2*(*tm1489*), and *tdp-1*(*ok803*) worms. Loss of HPL-2 resulted in a 6.8-fold reduction in *gsa-1* transcripts, and loss of TDP-1 resulted in a 1.5-fold reduction. Red histograms display RIP-seq recovery of dsRNA transcripts (normalized to total RNA) for wild-type, *hpl-2*(*tm1489*), and *tdp-1*(*ok803*) worms. Note the large increases in dsRNA representation of *gsa-1* transcripts with HPL-2 or TDP-1 loss. In panels B and C, error bars show SEM.

If HPL-2 acts in concert with TDP-1 to modulate RNA metabolism, a strong prediction is that loss of HPL-2 should replicate transcriptome changes we previously observed in the *tdp-1* deletion strain. We therefore used transcriptome sequencing (RNA-seq) to characterize the transcriptome of a strain containing the *hpl-2* null deletion allele *tm1489* under the same conditions as those used previously to characterize the *tdp-1*(*ok803*) transcriptome (15). Loss of HPL-2 had dramatic effects on transcript abundance, with 50% of transcripts assayed showing significant changes (Data Set S4). Transcripts with significant changes in the *tdp-1*(*ok803*) data were significantly overrepresented in the *hpl-2*(*tm1489*) data (among genes expressed in both data sets), with 72% of significant genes from the *tdp-1* list present on the *hpl-2* list (hypergeometric *P* = 1.7 × 10^−25^) (Fig. 6A). Importantly, there was an even more significant overlap among genes underexpressed in *tdp-1* mutants and those underexpressed in *hpl-2* mutants (75% codecreased) (*P* = 3.5 × 10^−62^; chi-square test), supporting the idea that *tdp-1*-dependent HPL-2 association functions to maintain gene expression. To validate the *hpl-2*(*tm1489*) RNA-seq data, quantitative reverse transcription-PCR (RT-PCR) was performed on independent RNA preparations from *hpl-2* and *tdp-1* mutants, targeting genes with reduced abundance based on the RNA-seq analysis. Coordinate reduction was observed in both strains as well as an *hpl-2*(*tm1489*); *tdp-1*(*ok803*) double mutant for 9/10 genes tested (Fig. 6B). Illustrating the relationship between chromatin association and transcript abundances, Fig. 6C shows TDP-1 ChIP sequencing (ChIP-seq) (green), HPL-2 ChIP-seq (blue), and RNA-seq (black) data for a representative gene, *gsa-1*. For wild-type animals, ChIP-seq experiments identified significant TDP-1 and HPL-2 binding peaks in intron 2 of this gene. Deletion of the *tdp-1* gene resulted in complete loss of the intron 2 HPL-2 binding peak and a significant reduction of *gsa-1* poly(A) transcripts (1.54-fold). Deletion of *hpl-2* similarly, and more dramatically, reduced *gsa-1* transcript accumulation (6.8-fold).

### Loss of HPL-2 results in accumulation of dsRNA transcripts.

Loss of TDP-1 results in a large accumulation of double-stranded RNA (dsRNA), which was previously quantified by a RNA immunoprecipitation (RIP-seq) experiment using the J2 monoclonal antibody to specifically immunoprecipitate dsRNA (15). We employed the same protocol to assay dsRNA in *hpl-2*(*tm1489*) mutants. For *tdp-1*(*ok803*) mutants, 89% of transcripts in the dsRNA pool were more abundant than those in the wild-type dsRNA pool. We found for *hpl-2*(*tm1489*) mutants that 84% of dsRNA transcripts followed this pattern, indicating a similarly strong enrichment for increased dsRNA formation. Over half the transcripts (784/1,419 transcripts) with increased dsRNA enrichment in *tdp-1*(*ok803*) mutants also showed increased dsRNA enrichment in *hpl-2*(*tm1489*) mutants, which is a highly significant overlap (hypergeometric *P* = 1.5 × 10^−144^). This pattern is also reflected for the *gsa-1* locus: deletion of either *tdp-1* or *hpl-2* led to a significant increase in double-stranded transcripts corresponding to the *gsa-1* gene (Fig. 6C, data in red).

Repetitive element (RE) transcripts contribute significantly to the dsRNA pool in C. elegans and are highly enriched in the dsRNA pool of *tdp-1*(*ok803*) mutants [471/477 RE transcripts with altered dsRNA representation are increased in *tdp-1*(*ok803*) mutants (15)]. Similarly, RE transcripts were preferentially enriched in the dsRNA pool of *hpl-2*(*tm1489*) mutants (424/478 significantly altered transcripts were enriched) (Data Set S5). Importantly, 300 of the RE transcripts with altered dsRNA representation in *hpl-2*(*tm1489*) mutants overlap significantly with altered transcripts in *tdp-1*(*ok803*) mutants, with a very high concordance (96%).

### Independent effects of TDP-1 and HPL-2 on alternative splicing.

Loss of TDP-1 results in changes in alternative splicing (15), as observed for TDP-43 knockdown (44). To determine if HPL-2 acts downstream of TDP-1 to modulate alternative splicing, we characterized splicing changes in *hpl-2*(*tm1489*) mutants. Using the rMATS algorithm, we detected splicing changes in 1,563 genes in *hpl-2*(*tm1489*) mutants (Data Set S6), a large fraction of which (602/1,563 genes) also had altered splicing in *tdp-1*(*ok803*) mutants. Unexpectedly, the majority of splicing changes in the *hpl-2* deletion mutants were not concordant with those observed in *tdp-1*(*ok803*) mutants. In fact, 74% of shared altered splicing events in *tdp-1*(*ok803*) and *hpl-2*(*tm1489*) mutants were discordant. Similarly, *tdp-1*(*ok803*) genes with altered splicing displayed no consistent pattern of increased or decreased HPL-2 binding. We concluded that altered splicing in *tdp-1*(*ok803*) mutants does not result from changes in HPL-2 chromatin association.

## DISCUSSION

Our previous work demonstrated that TDP-1 limits dsRNA accumulation. As dsRNA is the source of small interfering RNAs (siRNAs), we investigated whether loss of TDP-1 altered RNA interference (RNAi) in C. elegans. Indeed, we found that loss of TDP-1 sensitizes C. elegans to exogenous RNAi. This effect is due to enhanced efficiency of nuclear RNAi (transcriptional gene silencing), as it is blocked by a loss-of-function mutation in *nrde-3*, a critical component of the nuclear RNAi machinery. The specific enhancement of nuclear exo-RNAi caused by loss of *tdp-1* makes it unlikely that exo-RNAi efficiency is increased in the *tdp-1* mutant simply because this mutation increases the accumulation of dsRNA, as this would presumably have an impact on both cytoplasmic and nuclear RNAi. Increased efficiency of exo-RNAi is also unlikely to be due to a defect in endo-siRNA biogenesis, as deep sequencing of small RNAs in *tdp-1*(*ok803*) mutants did not show decreased abundances of siRNAs directed against most genes or NRDE-3 target genes (Fig. 3A) but, instead, an increase in siRNA levels. Our results differ from those reported by Krug et al., who found reduced siRNA accumulation and RNAi responses in flies overexpressing hTDP-43 (15). If the fly model results in reduced overall TDP-43 activity due to protein aggregation, the discrepancy between that study and ours may be the result of system-specific differences: TDP-1 lacks the C-terminal low-complexity domain present in fly and mammalian TDP-43, NRDE-3 is a worm-specific factor, and the increased RNAi response we characterized was elicited with a cytoplasmic dsRNA trigger, whereas Krug et al. expressed a nuclear hairpin. Alternatively, as hTDP-43 overexpression in Drosophila leads to cytoplasmic aggregation, effects on siRNA biogenesis and function may be due to a toxic gain-of-function mechanism unrelated to effects observed for worm *tdp-1* deletion. However, we favor the interpretation that reduced siRNA accumulation in the fly model results from an effective increase in TDP-43 activity due to hTDP-43 overexpression, which would be consistent with a loss of TDP-1 increasing siRNA levels in the worm model. This interpretation is supported by the observation that knockdown of the Drosophila TDP-43 homolog, TBPH, partially rescues motor neuron deficits resulting from hTDP-43 overexpression (7).

Why does loss of TDP-1 lead specifically to enhanced nuclear exo-RNAi? While we cannot rule out the possibility that TDP-1 is a novel negative regulator of nuclear exo-RNAi, we favor the idea that loss of TDP-1 increases the availability of chromatin-modifying proteins that contribute to nuclear exo-RNAi. In support of this, we show that TDP-1 coimmunoprecipitates with HPL-2 in the absence of *eri-1*-dependent endo-siRNAs and nuclear NRDE-3 (Fig. 4A), supporting a nuclear RNAi-independent interaction between TDP-1 and HPL-2. By analogy with the observation that mutations (e.g., in *eri-1* and *rrf-3*) that reduce cytoplasmic endo-RNAi can lead to increased availability of shared factors to the cytoplasmic exo-RNAi pathway, we hypothesize that *tdp-1* deletion enhances nuclear RNAi by increasing the amount of available HPL-2. Supporting this idea, we show by ChIP assay that TDP-1 maintains localization of HPL-2 in hundreds of active genes but also indirectly limits the HPL-2 ChIP signal for siRNA target genes (Fig. 4C). These results are consistent with the existence of two redundant or parallel pathways that maintain HPL-2 association in C. elegans, namely, an endo-RNAi-dependent NRDE-3/HPL-2 pathway and an endo-RNAi-independent TDP-1/HPL-2 pathway. This parallel pathway model is supported by our finding that *nrde-3*(*gg66*); *tdp-1*(*ok803*) double mutants show synergistic phenotypes (Fig. 3C to E), including temperature-sensitive, maternal-effect sterility, which is a phenotype of *hpl-2* null mutants (46). Interestingly, while *hpl-2* has also been shown to interact in the synthetic multivulva (synMuv) pathway, as a class B gene (32), we did not observe a synMuv phenotype in *tdp-1*(*ok803*) mutants in combination with either class A or class B synMuv genes, suggesting that HPL-2 function in vulva formation is independent of TDP-1.

HP1 homologs localize to active genes and coimmunoprecipitate with elongating RNA Pol II (26), but how the specificity of HP1 localization to only certain genes is established is unknown. Here we provide evidence that the localization of HPL-2 to specific genes can be mediated by TDP-1. We show that TDP-1 and HPL-2 associate *in vivo*, independent of RNA (Fig. 4A), and that in the absence of TDP-1, HPL-2 localization is reduced in genes bound by TDP-1 as well as being mislocalized globally. To our knowledge, this is the first example of an RNA binding protein known to cotranscriptionally bind chromatin acting to recruit an HP1 homolog to specific genes. As TDP-1's association with chromatin is dependent on RNA (15), our data support a model in which TDP-1 recruits HPL-2 and/or maintains HPL-2 association with nascent transcripts. While we do not know the precise mechanism behind TDP-1-mediated recruitment of HPL-2, we speculate that TDP-1 likely binds the nascent transcript through its RNA recognition motifs and binds HPL-2 directly via protein-protein interaction. Interestingly, HP1α, a mammalian homolog of HPL-2, was recently shown to form phase-separated liquid droplets (47, 48), as previously demonstrated for the TDP-1 ortholog TDP-43 (49). We speculate that TDP-1 and HPL-2 may interact as components of a nuclear “membraneless organelle” analogous to cytoplasmic RNA granules. HPL-2 may also directly bind transcripts, as HP1 homologs can bind RNA through their hinge domain (50). However, HP1 binding to RNA is not known to be sequence specific, so TDP-1 may provide this specificity. In support of this idea, our analysis of HPL-2 consensus binding sites revealed that the canonical TDP-1/TDP-43 binding site, (TG)_*n*_, is also highly enriched among HPL-2 binding motifs. Importantly, the (TG)_*n*_ binding motif was not enriched in HPL-2 ChIP assays of *tdp-1* mutants, indicating that HPL-2's specificity for (TG)_*n*_ is dependent on TDP-1. Interestingly, TDP-1 also facilitated HPL-2 association with (AG)_*n*_ repeats. While TDP-1 is not known to bind (AG)_*n*_ repeats, the consensus binding motif of another splicing factor, SFSR1, does contain (AG)_*n*_ repeats (51), and this factor was previously demonstrated to immunoprecipitate with human HP1 (26, 29). Therefore, TDP-1 may recruit HPL-2 through interaction with other splicing factors, such as SR proteins. While it is possible that TDP-1 shows a novel specificity for (AG)_*n*_ repeats in worms, we disfavor this idea, as *in vitro* assays indicate that TDP-1 does not have affinity for dinucleotide repeats other than (UG)_*n*_ (18). Regardless, maintaining localization of HPL-2 to nascent transcripts appears to be a major function of TDP-1, as over half the regions that we previously identified as cotranscriptionally bound by TDP-1 showed a significant change in HPL-2 association in the *tdp-1*(*ok803*) mutant, with the majority of regions losing HPL-2 localization.

RNA-seq analysis of the *tdp-1*(*ok803*) and *hpl-2*(*tm1489*) deletion strains revealed a highly significant overlap in transcript abundance changes, although loss of HPL-2 appears to have a more dramatic effect on the global transcriptome. Similarly, many transcripts found to have increased dsRNA structure in *tdp-1*(*ok803*) mutants also had increased dsRNA structure in *hpl-2*(*tm1489*) mutants, including transcripts from repetitive elements. We interpret these observations to indicate that (i) the proximal cause of a significant fraction of the *tdp-1*(*ok803*) transcriptome changes we previously identified may in fact be due to altered HPL-2 chromatin association and (ii) HPL-2 also has additional, TDP-1-independent roles in RNA metabolism. It would be of significant interest to know the degree to which HP1 isoforms play a role in the transcriptome changes identified in TDP-43 knockdown (44) or loss-of function (52) models.

What is the molecular function of TDP-1-mediated recruitment of HPL-2? It was previously shown that TDP-43 (and presumably TDP-1) can act as an RNA chaperone to control the accumulation of double-stranded RNA (15). Perhaps HPL-2 assists TDP-1 in limiting potential RNA structure via this activity. Importantly, homologs of both TDP-43 and HP1 have been shown to coimmunoprecipitate with repetitive RNA (14, 27), further supporting a common function for these two proteins on structured transcripts. While HP1's primordial function may be the compaction of DNA, perhaps HP1 can compact other nucleic acids as well, as suggested previously (28). Conceivably, HP1 helps to compact and package nascent transcripts in order to maintain the structural limitations imposed by TDP-1. Alternatively, TDP-1 may recruit HPL-2 to initiate a chromatin signature in the DNA that signals the presence of repetitive, structured RNA, possibly altering transcription elongation to allow for correct processing of this RNA. Either way, loss of TDP-1-dependent HPL-2 association is correlated with both decreases in transcript abundance and increases in dsRNA structure, indicating that recruitment of HPL-2 or maintenance of HPL-2 association is important for TDP-1-mediated RNA processing.

TDP-43, the human ortholog of TDP-1, is centrally involved in ALS/FTD. Significant transcriptome changes are observed in animal models of TDP-43 pathology (1, 15, 44, 53, 54) as well as in brains of ALS patients (55, 56), including changes in the abundance of neuron-specific transcripts and repetitive element transcripts. Overexpression of hTDP-43 in Drosophila increases levels of the *gypsy* retrotransposon element, resulting in a reduced life span and in neurodegeneration. As the Drosophila HP1 homolog binds retrotransposon-derived RNA (27) and its knockdown reduces the life span (57), it is possible that some of the hTDP-43 overexpression effects are mediated through dysregulated HP1 recruitment. Regardless, our data suggest that changes in chromatin association of HP1 proteins homologous to HPL-2 may underlie potentially pathology-related transcriptome alterations.

## MATERIALS AND METHODS

### Strains and genetics.

Maintenance and growth of worms were performed as described previously (58), and all strains were raised at 20°C unless noted otherwise. Deletion alleles were generated by the Gene Knockout Consortium (University of British Columbia, Vancouver, Canada). All transgenic strains used in this study were created by gonad injection and subsequent integration of the DNA array. Genetic construction of deletion alleles and mutations was followed by single-worm PCR or phenotyping. Strains used or created in this work are shown in Table 1.

**TABLE 1:** Strains used or created in this work

Strain	Genotype	Source
CL6311	*tdp-1*(*ok803*)*II* X8 outcross to CGC-N2B	Original allele from CGC, University of Minnesota, MN; outcrossed in-house
XQ8	*tdp-1*(*ok781*)*II* X5 outcross	Parker lab
YY158	*nrde-3*(*gg66*)*X*	CGC, University of Minnesota, MN
CL6185	FLAG::NRDE-3::GFP	Kennedy lab
PD4251	*dpy-20*(*e1282*); *ccIs4251* (P*myo-3*::GFP)	CGC, University of Minnesota, MN
CL6194	*tdp-1*(*ok781*)*II*; *ccIs4251* (P*myo-3*::GFP)	This study
CL6195	*tdp-1*(*ok803*); *ccIs4251* (*Pmyo-3*::GFP)	This study
CL6263	*tdp-1*(*ok803*)*II*; *eri-1*(*mg366*)*V*	This study
CL6424	*tdp-1*(*ok803*); *nrde-3*(*tm1116*)	This study
GR1373	*eri-1*(*mg366*)*V*	CGC, University of Minnesota, MN
CL2643	P*snb-1*::TDP-1	This study
CL6491	*tdp-1*(*ok803*); *nrde-3*(*gg66*)	This study
PFR40	*hpl-2*(*tm1489*)	CGC, University of Minnesota, MN

### Microscopy.

GFP fluorescence images were acquired with a Zeiss Axiophot microscope equipped with digital deconvolution optics (Intelligent Imaging Innovations), and image brightness and contrast were digitally adjusted in Photoshop.

### Thrashing assay.

Liquid thrashing assays were performed as described previously (59), using synchronized 1-day-old adults grown at 25°C. Thrashes were counted for 30 s by hand under a dissecting microscope.

### Brood assay.

For each assay, 5 individual L4 worms of each indicated genotype were singly picked to a small plate spotted with OP50 bacteria. Each day, worms were moved to fresh plates and the progeny counted until no more progeny were laid. The total brood for each animal was determined by adding the numbers of progeny laid on all days. The assay was done twice in triplicate.

### Extracts.

Worms were broken by bead beating (mini-bead beater; Biospec Products) in homogenization buffer (10 mM KCl, 1 mM dithiothreitol [DTT], 10 mM Tris-HCl [pH 8.0], 50 mM sucrose, 0.05% Nonidet P-40, 1 mM Complete protease inhibitor [Sigma]) at a 1:1 ratio of 0.1-mm glass beads to packed worm volume for 3 cycles of 30 s each at 5,000 rpm. Extracts were used immediately for immunoprecipitations, with equal concentrations of extract (as determined by protein quantification) added to IP mixtures. ChIP extracts were prepared as described previously (15).

### Immunoblotting.

For immunoblotting, 10 to 20 μg of protein per sample or equal volumes of immunoprecipitated protein samples were run at 180 V in NuPAGE 4 to 12% Bis-Tris gels (Invitrogen), using morpholineethanesulfonic acid (MES)-SDS running buffer (Invitrogen). The gels were transferred to 0.45-μm supported nitrocellulose membranes (GE Osmonics) by use of 20% methanol, 39 mM glycine, and 48 mM Tris base. Transfer conditions were 35 V for 70 min. Prestained Rainbow size markers (RPN755 and RPN800; Amersham Biosciences) were used to size bands. Blots were blocked in Tris-buffered saline (TBS)–Tween (100 mM Tris, pH 7.5, 150 mM NaCl, 0.1% Tween 20) plus 5% milk. Blots were probed with a primary mouse monoclonal antibody against green fluorescent protein (1:2,000; Invitrogen), rabbit polyclonal anti-TDP-1 (1:1,000; made in-house), and anti-HPL-2 (1:2,000; generously provided by the Palladino lab). For reprobing of blots to assay loading levels, a C. elegans-specific Cstf64 rabbit polyclonal antibody (generously provided by the Blumenthal lab) was diluted 1:7,500 in blocking solution. Horseradish peroxidase (HRP)-conjugated secondary antibodies (1:10,000 goat anti-rabbit antibody, light chain specific, or 1:1,000 goat anti-mouse antibody; Sigma) were developed with ECL Plus reagent (Amersham).

### Protein immunoprecipitations.

Twenty microliters of protein A magnetic beads (Dynabeads; Invitrogen) or anti-FLAG magnetic beads (Invitrogen) was washed, blocked, and bound by antibody (Dynabeads only) at a ratio of 10 μl/IP for anti-TDP-1 (made in-house) or 3 μl/IP for anti-HPL-2 (kindly supplied by the Palladino lab), and 100 μg of protein was added to each IP mixture. RNase-treated extract was incubated with 1 to 5 μl of RNase T1 (single-stranded RNA specific) and RNase V1 (double-stranded RNA specific) for 30 min (room temperature) prior to addition to beads. Tubes were rocked at 4°C for 2 h. The supernatant was removed, and beads were washed three times in IP buffer (15), moved to a new tube, and washed two more times with buffer. Immunoprecipitated protein was removed from beads by boiling in SDS protein loading buffer for 5 min followed by a light spin and then was frozen at −20°C or loaded immediately onto an SDS-PAGE gel.

### ChIP assay.

ChIP was performed as described previously (60). A cross-linked extract was resuspended and sonicated with a Virsonic digital 600 sonicator with a microtip to generate DNA fragments of approximately 500 bp. Samples were sedimented and divided into four 1-ml aliquots. For each 1-ml aliquot (2 mg protein per sample), 100 μl of protein A Dynabeads (Invitrogen) conjugated with 3 μl HPL-2 antibody (kindly provided by the Palladino laboratory) was added. After overnight incubation at 4°C, beads were washed and DNA was eluted. Ten microliters of proteinase K (20 mg/ml) was added to the eluted fraction and incubated at 55°C for 2 to 3 h. The tubes were then transferred to 65°C overnight to reverse cross-links. DNA was purified using a Qiagen column (Qiaquick) and eluted twice with 30 μl of water. TDP-1 ChIP-seq data were obtained by using accession number GSE61581.

### Quantitative RT-PCR.

Quantitative RT-PCR was performed on two independent biological replicates of each indicated strain. Total RNA was isolated using TRIzol, and oligo(dT)-primed cDNA was synthesized by use of SuperScript IV (Invitrogen) and amplified using the primers shown in Table 2. The signal was normalized to the signal of the control transcript *pmp-3*, which shows no change in expression in mRNA-seq experiments between wild-type, *tdp-1*(*ok803*), and *hpl-2*(*tm1489*) strains. The fold changes in signal between the wild-type and mutant strains were calculated using the ΔΔ*C_T_* method.

**TABLE 2:** Primers used for quantitative RT-PCR verification of abundance changes in the *tdp-1*(*ok803*), *hpl-2*(*tm1489*), and double mutants

Gene	Primer sequence (5′–3′)
Y39E4B.6	GACCGAGAATCGACACCTTC
	ACTGGTGATTTCGACTGTCC
K11D2.4	CCGGAACAACAGCAGAATCA
	GGCCGAGAAGTGACGAAAT
*atx-2*	GGTGTTCCTGGACAGATGTATG
	GTGGCTGCTTCTCACGATAC
*gsa-1*	ACAACCGCTGGCTGAAA
	TATCGAACACCGCGTCATTAG
*pqn-42*	AGATTCGCGTCCATCATCAG
	ACATTTCCTCCACACCAACA
F57F4.4	ATCCATCACAGAATTGGTCTCC
	TGATCCAAACAGAGGATCTCAAC
*let-805*	AAGCTTCGCGCCACTAAT
	GTGAACCTCGTTTGGAGACA
*unc-52*	CATGGAGCCATTCGACTTCT
	CAACATCGGACCGTCTCTTAC
*gei-3*	GATGTCGCTGCGGCTATTA
	TCGCAACCATCATCGGATAC
Y48E1A.1	CCCGAACACTCAACAAGACA
	GATTGCGAACGTCGGAAGTA
*pmp-3*	GTTCCCGTGTTCATCACTCAT
	ACACCGTCGAGAAGCTGTAGA

### RNA isolation, cDNA library preparation, and high-throughput sequencing.

For all *tdp-1*(*ok803*) analyses performed, we used previously reported RNA-seq data (15). Information on RNA isolation, cDNA preparation, and sequencing can be obtained from the previous report. For small RNA libraries, 20 μg of DNase-treated total RNA was brought up in 2× formamide loading buffer, boiled for 5 min at 95°C, loaded into a 12% denaturing polyacrylamide gel, and electrophoresed. Small RNAs corresponding to a size range of 18 to 30 nucleotides (nt) were excised from the gel. RNA was extracted from the gel slice by adding 700 μl 0.3 M NaCl and 1 μl RNaseout, rocked overnight at 4°C, and spun through a Spin X cellulose acetate filter. Isolated RNA was precipitated with isopropanol, washed, and dissolved in 12 μl RNase-free H_2_O. RNA was treated with shrimp alkaline phosphatase (NEB) for 30 min at 37°C in company-supplied buffer, phenol-chloroform extracted, ethanol precipitated, and brought up to 30 μl in RNase-free H_2_O. RNA was then incubated at 37°C for 30 min with polynucleotide kinase (NEB) in DNA ligase buffer (containing ATP), with the addition of 1 μl of RNaseout (Invitrogen). RNA was phenol-chloroform extracted, precipitated with isopropanol, washed, and dissolved in 12 μl RNase-free H_2_O. Library construction was done using an Illumina TrueSeq small RNA sample prep kit.

For *hpl-2*(*tm1489*) analyses, RNAs for poly(A) and total RNA sequencing libraries were extracted from whole animals by TRIzol extraction. Genomic DNA was removed using Turbo DNase (Invitrogen). Poly(A)-selected, single-ended, 125-bp strand-specific libraries were prepared by the UCCC Genomics Core (Aurora, CO) by use of a TruSeq stranded mRNA library prep kit (Illumina). For J2-IP analyses, RNAs were recovered from young adult worms (three biologically independent lysates) by immunoprecipitation with the J2 antibody, and RNAs as well as input material (as a loading control) were converted into strand-specific total RNA libraries by use of V2 Scriptseq (Epicenter) kits following the manufacturer's instructions, except that reverse transcription was done with SuperScript III (Invitrogen), using incrementally increasing temperatures from 42 to 59°C to allow for transcription though structured RNAs. rRNA was not removed from J2-IP RNA samples. Immunoprecipitated DNA from ChIP samples was converted into sequencing libraries by use of a ChIP-seq DNA sample prep kit. Cluster generation and sequencing were performed on an Illumina HiSeq 2500 platform. The reads were demultiplexed and converted to FASTQ format by use of CASAVA software from Illumina.

### Mapping and analysis of small RNAs.

Small RNA reads were trimmed to remove adaptor sequences and filtered to allow only 1 base with a quality score below 20 and a minimum sequence length of 15 bp. Reads passing the filter were mapped to the WS220 genome by use of Bowtie1 (61), allowing 1 mismatch in a 15-bp seed. Reads mapping to multiple locations were removed. For quantification of antisense siRNAs, mapped SAM files were filtered to remove reads with at least 15 bp of overlap with annotated miRNAs and piRNAs (WS220) (www.wormbase.org). Quantification of antisense siRNAs mapping to genes was done by separating SAM files into positive and negative reads and counting the numbers of siRNAs mapping to defined gene intervals. Only siRNAs mapping antisense to the annotated gene were counted. Expression differences between the wild-type and *tdp-1* mutant strains were calculated using DESeq software, with cutoffs of an FDR of <0.05% and a >2-fold change.

### Sequence alignment, gene expression quantification, and J2-IP analysis (RNA-seq).

Low-quality bases (*q* < 10) were trimmed from the 3′ ends of reads. Adaptor sequences and reads shorter than 40 nucleotides were removed. Reads were aligned to the C. elegans WS220 genome by use of TopHat2 (v2.0.14), with the following parameters: -b2-very-sensitive -i 30 -I 5000 -read-edit-dist 3 -N 3 -read-realign-edit-dist 0 -p 10 -segment-length 25 -segment-mismatches 2 -no-coverage-search -min-coverage-intron 30 -max-coverage-intron 5000 -min-segment-intron 30 -max-segment-intron 5000. For poly(A)-selected RNA-seq reads, uniquely mapped reads were used as input to obtain gene counts by using the WS220 annotation with Rsubread (v 1.18.0) (62). Differential expression of genes was determined using DESeq2 (v1.14.1) (63). Significance of differences was assigned to genes with FDR values of <10%. The bioinformatics protocol for identification of transcripts enriched by J2-IP was previously explained in detail (15). RNA-seq data for the *tdp-1*(*ok803*) mutant were taken from the data available under accession number GSE61581. Details related to all sequencing experiments done in this work can be found in Table 3.

**TABLE 3:** Deep sequencing performed for this work

Strain	Type of sequencing	No. of samples^*a*^	Worm stage	Temp (°C)
N2	mRNA	2	L4	20
*hpl-2*(*tm1489*) strain	mRNA	3	L4	20
N2	Total RNA	1	Young adult	15
*hpl-2*(*tm1489*) strain	Total RNA	3	Young adult	15
N2	J2-RIP-seq	3	Young adult	15
*hpl-2*(*tm1489*) strain	J2-RIP-seq	3	Young adult	15
N2	HPL-2 ChIP-seq	2	Young adult	20
*tdp-1*(*ok803*) strain	HPL-2 ChIP-seq	2	Young adult	20
N2	Genomic DNA	1	Young adult	20
*tdp-1*(*ok803*) strain	Genomic DNA	1	Young adult	20
N2	Small RNA	2	Young adult	20
*tdp-1*(*ok803*) strain	Small RNA	2	Young adult	20

a All sequence experiments with >1 sample were biological replicates.

### J2-IP repetitive element analysis.

Trimmed and filtered reads from total RNA and J2-IP samples were used as input into the RepEnrich program to determine repetitive element expression levels (64). The WS220 repeatmasker file required by RepEnrich was downloaded from http://repeatmasker.org. The suggested parameters for running RepEnrich on single-end reads were used. The commands are available at https://github.com/nskvir/RepEnrich.

To determine if repetitive elements were significantly enriched in the J2-HPL-2 samples, we used DESeq2. Repeat counts were normalized by obtaining the difference between total mapped reads and the number of reads aligning to rRNA. DESeq2's likelihood ratio test was used, and a minimum mean expression value of 20 and an adjusted *P* value of <0.10 were used to determine repeat significance. J2-IP RNA-seq data for the *tdp-1*(*ok803*) mutant were taken from the data available under accession number GSE61581.

### Mapping and analysis of HPL-2 by ChIP-seq.

Chromatin immunoprecipitation sequencing (ChIP-seq) and genomic reads were trimmed and filtered in the same manner as that described above for RNA-seq data. ChIP-seq and genomic reads were uniquely mapped to the WS220 genome by use of Bowtie1, allowing 2 mismatches in a 45-bp seed. MACS, version 1.4.2, was used to identify significant peaks (*q* < 0.05) for the wild-type and *tdp-1*(*ok803*) HPL-2 ChIP-seq samples, using the corresponding [N2 and *tdp-1*(*ok803*)] genomic sequencing samples as background controls. Parameters for MACS peak calling used for both the wild-type and *tdp-1*(*ok803*) strains were as follows: callpeak -t Strain_HPL2.bam -c Strain_genomic_control.bam -B -g ce -keep-dup 3 -n Strain_HPL2 -m 2 50. To call differentially represented peaks between wild-type and *tdp-1*(*ok803*) samples, we used the Bioconductor program Diffbind (version 1.10.2). To briefly elaborate, we first input the significant MACS peak calls for all samples, along with the corresponding HPL-2 ChIP-seq and genomic alignment files, into Diffbind. Diffbind identified a consensus set of peaks found in our MACS peak calls, with the requirement that at least 2 samples contain the peak. Read counts for each consensus peak were generated. Genomic read counts for the corresponding consensus peaks were subtracted from those for their corresponding samples (i.e., N2_HPL-2 − N2_genomic). Differential analysis of the peak counts between the wild-type and *tdp-1*(*ok803*) strains was performed using DESeq. An FDR cutoff of <10% was used to determine the significance of differences.

To determine the overlap of HPL-2 peaks with repetitive element sequences, consensus peaks were derived from our biological replicate wild-type HPL-2 ChIP-seq reads by use of Diffbind. The consensus wild-type HPL-2 peaks were intersected with the CE10 repeatmasker file (http://repeatmasker.org), and one repeat overlap per peak was kept if at least one nucleotide overlapped.

### Permutation tests.

Permutation tests were done to compare called HPL-2 ChIP-seq peaks for wild-type animals to ChIP-seq peaks previously identified by McMurchy et al. (31). HPL-2 broad and concave peaks were both used for the analysis. The Bioconductor package regioneR (65) was used to compare the peaks identified by McMurchy et al. to peak calls in this report. Peak sets were shuffled 1,000 times for each of the permutation tests. The widths of the peaks and total number of peaks from the tested sets were used for the widths of the random peaks. The entire WS220 genome (including repeat regions), except for the mitochondrial genome, was available for pulling of the random peaks. To test the significance of the overlap between our wild-type HPL-2 ChIP peaks and repetitive elements in the genome, we used the same parameters as those described above with the ce10.fa.out repeat location file from http://repeatmasker.org. The following options were used for all three permutation tests: permTest(ntimes = 1000, randomize.function = randomizeRegions, evaluate.function = numOverlaps, genome = “WS220”, count.once = true).

### HPL-2 ChIP-seq motif analysis.

We first derived consensus peak sets for wild-type and *tdp-1*(*ok803*) replicate HPL-2 ChIP-seq samples by using MACS. The consensus peak sets were then analyzed using HOMER, a software suite for ChIP-seq analysis and motif discovery. The UCSC ce10 genome was used for the HOMER motif search, with the option “-size 200.”

### Analysis of alternative splicing.

RNA-seq reads were mapped by use of the STAR algorithm to the Wormbase WS258 genome and transcript annotation (66). Multisample 2-pass mapping was employed to increase novel junction discovery, and alignment parameters were tuned to account for the relatively small average intron size in C. elegans. The STAR parameters used were as follows: -alignIntronMin 10 -alignIntronMax 5000 -outSJfilterIntronMaxVsReadN 10 20 40 -outSJfilterCountTotalMin 5 3 3 3 -outSJfilterCountUniqueMin 3 2 2 2. rMATS (v3.2.5) software was used to identify alternative splicing (AS) events, which corresponded to five types of AS patterns (67). AS events were identified as significant if the FDR was <0.1.

### RNA interference.

Single colonies from the Ahringer library (68) were inoculated and grown using previously described methods (69). Bacteria were allowed to grow to a final optical density of ∼0.4. For dilution series, the bacteria were serially diluted using neutral carrier bacteria [E. coli HT115(DE3) containing an empty L4440 vector] grown to the same optical density. In each case, 250 μl of RNAi food was seeded onto a 10-mm or 3-mm worm growth plate containing solid worm growth agar freshly spread with 1 mM isopropyl-β-d-thiogalactopyranoside (IPTG; LabScientific) and 0.11 mM ampicillin (AMP; Research Products International Corp.). IPTG and AMP were allowed to disperse through the solid agar for 1 h, and then the plates were immediately spotted with RNAi food. Bacteria were grown overnight at room temperature. Worms of the desired strain were placed onto each seeded plate at 20°C as L4 worms and allowed to lay eggs for 16 h. Parents were removed, and RNAi phenotypes were observed in hatched progeny. For heritable RNAi experiments, synchronized wild-type and *tdp-1*(*ok803*) animals expressing a muscle-specific GFP transgene were treated with GFP feeding RNAi for 1 generation. Embryos from these animals were collected and hatched on normal (OP50) bacteria. The resultant progeny were scored for GFP signals 2 days later, as L4 worms. The criteria applied to determine whether animals were affected by a given RNAi treatment were as follows: *unc-22* RNAi- and *unc-54* RNAi-treated animals were considered affected if the animals were unable to move away after prodding with a pick, *act-5* RNAi-treated animals were considered affected upon L2 arrest, and *unc-73* RNAi-treated animals were considered affected if they failed to back up and displayed a “kinked tail” upon prodding of the head region with a pick.

### Accession number(s).

The raw data generated in this study can be found in the Gene Expression Omnibus (GEO) database under accession number GSE100829.

## Supplementary Material

Supplemental material
