# Supplementary material for: The Caenorhabditis elegans Ortholog of TDP-43 Regulates the Chromatin Localization of the Heterochromatin Protein 1 Homolog HPL-2
Source: Mol Cell Biol. 2018 Jul 16;38(15):e00668-17. doi: 10.1128/MCB.00668-17 (PMC6048318; doi:10.1128/MCB.00668-17)
Supplement: Supplemental material [file supp_38_15_e00668-17__index.html]

Supplemental material 

# The Caenorhabditis elegans Ortholog of TDP-43 Regulates the Chromatin Localization of the Heterochromatin Protein 1 Homolog HPL-2

## Supplemental material

- Supplemental file 1 -

  Data Set S1 (Genes with statistically significant differences in antisense siRNA abundance between wild-type and mutant animals)

  XLSX, 82K
- Supplemental file 2 -

  Data Set S2 (Annotation of Diffbind results for HPL2 ChIP analysis)

  XLSX, 5.6M
- Supplemental file 3 -

  Data Set S3 (Overlap of TDP-1 and HPL-2 ChIP-seq peaks)

  XLSX, 1.3M
- Supplemental file 4 -

  Data Set S4 (Transcript abundance changes in HPL-2 deletion mutant)

  XLSX, 2.2M
- Supplemental file 5 -

  Data Set S5 (J2-enriched repeats in *tdp-1* and *hpl-2* mutants)

  XLSX, 125K
- Supplemental file 6 -

  Data Set S6 (Splicing alterations in HPL-2 deletion strain)

  XLSX, 2.7M
- Supplemental file 7 -

  Movie S1 (Wild-type animal thrashing in M9 buffer)

  MP4, 7.9M
- Supplemental file 8 -

  Movie S2 [*tdp-1*(*ok803*); *nrde-3*(*gg66*) mutant animal thrashing in M9 buffer)

  MP4, 5.0M
- Supplemental file 9 -

  Legends to Movies S1 and S2

  PDF, 39K
