# Supplementary material for: The Caenorhabditis elegans Ortholog of TDP-43 Regulates the Chromatin Localization of the Heterochromatin Protein 1 Homolog HPL-2
Source: Mol Cell Biol. 2018 Jul 16;38(15):e00668-17. doi: 10.1128/MCB.00668-17 (PMC6048318; doi:10.1128/MCB.00668-17)
Supplement: Supplemental material [file MCB.00668-17_zmb015181798s9.pdf]

Movie S1,S2: *tdp-1(ok803),nrde-3(gg66)* double mutant animals have movement defects

(S1,S2) Representative movies taken under magnification of wild type (**S1**) and *tdp-1(ok803),nrde-3(gg66)* (**S2**) animals thrashing in M9 buffer. Animals were filmed as 1-day old adults grown at 25^0 from embryo.
